# Overexpression of Transglutaminase from Cucumber in Tobacco Increases Salt Tolerance through Regulation of Photosynthesis

**DOI:** 10.3390/ijms20040894

**Published:** 2019-02-19

**Authors:** Min Zhong, Yu Wang, Yuemei Zhang, Sheng Shu, Jin Sun, Shirong Guo

**Affiliations:** 1 Key Laboratory of Southern Vegetable Crop Genetic Improvement, Ministry of Agriculture, College of Horticulture, Nanjing Agricultural University, Nanjing 210095, China; 2016204040@njau.edu.cn (M.Z.); ywang@njau.edu.cn (Y.W.); zym941128@163.com (Y.Z.); shusheng@njau.edu.cn (S.S.); jinsun@njau.edu.cn (J.S.); 2 Suqian Academy of Protected Horticulture, Nanjing Agricultural University, Suqian 223800, China

**Keywords:** TGase, photosynthesis, salt stress, polyamines, cucumber

## Abstract

Transglutaminase (TGase) is a regulator of posttranslational modification of protein that provides physiological protection against diverse environmental stresses in plants. Nonetheless, the mechanisms of TGase-mediated salt tolerance remain largely unknown. Here, we found that the transcription of cucumber *TGase* (*CsTGase*) was induced in response to light and during leaf development, and the CsTGase protein was expressed in the chloroplast and the cell wall. The overexpression of the *CsTGase* gene effectively ameliorated salt-induced photoinhibition in tobacco plants, increased the levels of chloroplast polyamines (PAs) and enhanced the abundance of D1 and D2 proteins. TGase also induced the expression of photosynthesis related genes and remodeling of thylakoids under normal conditions. However, salt stress treatment reduced the photosynthesis rate, PSII and PSI related genes expression, D1 and D2 proteins in wild-type (WT) plants, while these effects were alleviated in *CsTGase* overexpression plants. Taken together, our results indicate that TGase-dependent PA signaling protects the proteins of thylakoids, which plays a critical role in plant response to salt stress. Thus, overexpression of TGase may be an effective strategy for enhancing resistance to salt stress of salt-sensitive crops in agricultural production.

## 1. Introduction

Photosynthesis is the basic manufacturing process in plants; it can increase carbon gains and improve crop yield and quality [1]. Salinity, along with other environmental stresses such as drought and chilling, induces inhibition of photosynthetic activity by disruption of the chloroplast structure and reduction in CO_2_ assimilation [2]. The effects of environmental stresses on photosynthesis in cucumber have been extensively studied. We have described several defense systems that protect the photosynthetic apparatus by exogenous polyamines (PAs) application. For example, exogenous spermidine (Spd) delays chlorophyll degradation under heat stress, and the regulation of fatty acids and accumulation of PAs in thylakoid membranes are induced by exogenous putrescine (Put) under salt stress [3,4]. PAs are low molecular weight aliphatic amines; the biochemical properties of PAs are quite simple, but their regulation of processes is strikingly complex and wide processes [5]. The majority of PAs in higher plants are Spd, spermine (Spm), and their precursor Put, which derive from arginine in chloroplasts [6,7]. On the other hand, as the polycationic nature of PAs at physiological pH, PAs could be free molecules conjugated with organic acids or bound to negatively charged macromolecules such as proteins, nucleic acids, and chromatin through transglutaminases (TGases) enzymatic activity, stabilizing their structures [8,9]. These interactions are essential for the effects of PAs on plant cell growth and developmental processes and plant response to various stresses.

Moreover, PAs, as organic cations and permeant buffers, have been reported to protect the photosynthetic apparatus by regulating the size of the antenna proteins of light harvesting chlorophyll a/b protein complexes (LHCII) and the larger subunit of ribulose bisphosphate carboxylase-oxygenase during stresses, such as UV-B radiation and salt stress [10,11]. PAs are also synthesized and oxidized in chloroplasts, while the addition of PAs inhibits the destruction of thylakoids and prevents the loss of pigment during salt stress [12,13]. The levels of endogenous PAs are also related to chlorophyll biosynthesis and the rate of photosynthesis during stresses [14]. PA accumulation in the lumen promotes an increase in ATP and the electric field in *vivo* and in *vitro* [15]. In addition, endogenous PAs might be involved in the assembly of photosynthetic membrane complexes such as thylakoid membranes [16].

TGases are crucial factors of the thylakoid system but are often rather ignored. TGases catalyze proteins by establishing ε-(γ-glutamyl) links, then regulate proteins post-translational modification and the covalent binding of PAs to protein substrates [17,18]. TGases are widely distributed in microorganisms, animals, and plants. However, research on these enzymes in plants is more rarely reported than in animal systems, in which was detected for the first time in *Arabidopsis thaliana* the presence of only one gene, *AtPng1p*, which encodes a putative *N*-glycanase containing the Cys-His-Asp triad of the TGase catalytic domain and was expressed ubiquitously [19]. Although TGases are found in several organs in lower and higher plants, they are activated in a Ca^2+^ dependent manner and are involved in fertilization, abiotic and biotic stresses, senescence, and programmed cell death, under different light environment conditions, including natural habitats, but the function of TGases in chloroplasts has received the most attention [18,20]. The activity of TGases has been shown to be light sensitive, and some proteins of the photosystems (LHCII, CP29, CP26, and CP24) have been shown to be endogenous substrates of TGase in chloroplasts [21]. Meanwhile, TGase not only localized in the chloroplast grana and close to LHCII but also localized in the walls of the bulliform cells of leaves. Its activity was light dependent and its abundance depended on the degree of grana development [22]. Moreover, the expression and activity of TGase was involved in length of light exposure in maize [23].

In earlier works, we described the effects of salt and heat stresses on changes of free PA contents in leaves of cucumber and tomato [24,25]. Specifically, the content of PAs decreased under stress conditions, resulting in severe damage to photosynthetic organs such as chloroplasts, which were severely deformed into irregular shapes, and increased starch granules. TGase was induced by salt stress and involved in the protection of the photosynthetic apparatus [26]. In this article, we reveal that TGase has a positive role in PAs accumulation and induce the transcript of photosynthetic genes in chloroplast, which may play crucial roles in the regulation of photosynthetic organ stability. To our knowledge, this is the first report to show that the TGase positively regulates plant’s photosynthetic through accumulation of PAs to enhance salt tolerance.

## 2. Results

### 2.1. Expression Profile Analysis of TGase

**Figure 1:**
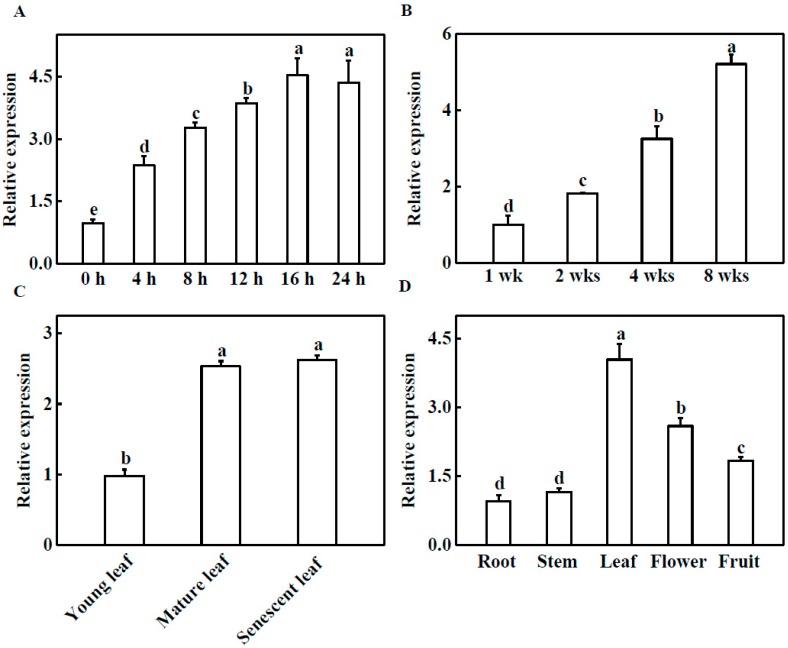
Expression profiles of *TGase*. (**A**) Light-induced *TGase* expression in cucumber. The cucumber seedlings were exposed to light 0, 4, 8, 12, 16 and 24 h and *TGase* transcript levels were analyzed by quantitative real-time PCR (qPCR). (**B**) Transcript levels of *TGase* in cucumber leaves at 1, 2, 4, 8 weeks of development. (**C**) Transcript levels of *TGase* in young, mature and old leaves of cucumber plants. (**D**) qPCR analysis of *TGase* transcript in roots, stems, leaves, flowers and fruit of cucumber. Each histogram represents a mean ± SE of four independent experiments (*n* = 4). Different letters indicate significant differences between treatments (*p* < 0.05) according to Duncan’s multiple range test.

To elucidate the molecular function of TGase, we analyzed the gene expression of *TGase* in cucumber. First, to investigate the effect of light on the expression of *TGase* in cucumber plants, we monitored the transcriptional levels of *TGase* in light-treated cucumber seedlings. The transcript level of *TGase* was gradually induced by light and reached the highest level at 16 h (Figure 1A). These results indicate that light plays a vital role in regulating *TGase* expression. To further explore the expression of *TGase* at different developmental stages, we investigated the transcript levels of *TGase* in leaves ranging from 1 to 8 weeks old by quantitative real-time PCR (qPCR). The expression level of *TGase* increased with the growth and development of the leaves (Figure 1B). *TGase* transcript levels in 4 and 8 week-old plants was significantly higher than those in 1-week-old plants (Figure 1B). Furthermore, *TGase* transcript levels increased during leaf development from young to mature leaves (Figure 1C). Some reports showed that TGase was widely present in plant tissue [17]. We extracted RNA from roots, stems, leaves, flowers, and fruits, and then analyzed the transcript levels of *TGase* in these tissues via qPCR. Our results also showed that *TGase* was present in all investigated tissues and was highly expressed in leaves and flowers, but minimally expressed in roots and stems (Figure 1D).

### 2.2. Immunolocalization of TGase Protein in Cucumber Leaves

**Figure 2:**
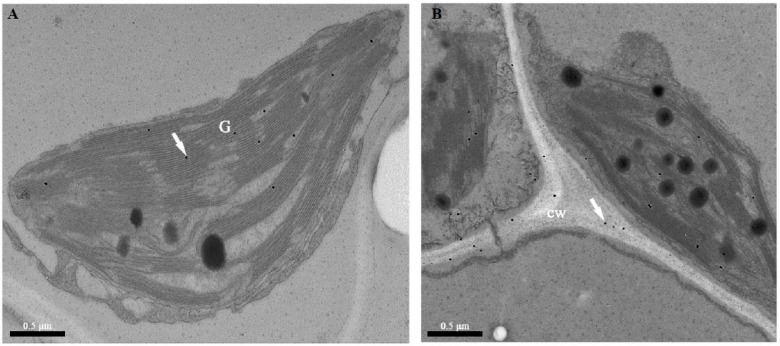
TEM immunolocalization of TGase in cucumber leaf chloroplasts using the monoclonal antibody (1:1000). (**A**) Signal in the granary of the chloroplasts. (**B**) Signal in the cell well. G, grana; cw, cell wall.

Subcellular immunolocalization in cucumber leaf mesophyll cells provided details on the presence of TGase. The signal was localized in the chloroplasts and near the chloroplast grana (Figure 2A). The presence of TGase spots in the cell wall was detected (Figure 2B). These were not significantly localized to TGase in other cell organelles.

### 2.3. Effects of TGase on the Biomass and Photosynthetic Characteristics of Transgenic Tobacco Lines

**Figure 3:**
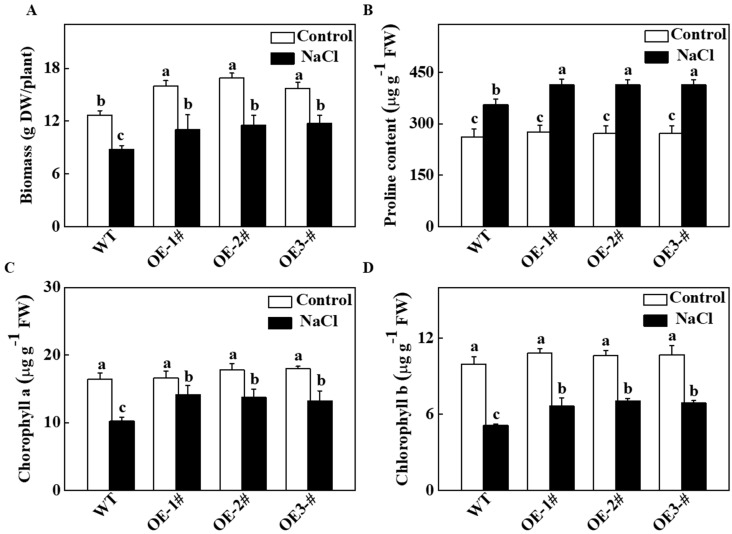
Effects of salt stress on biomass, proline and chlorophyll content in wild-type (WT) and *CsTGase*OE plants. (**A**) Biomass. (**B**) Proline content in leaves. (**C**,**D**) Chlorophyll a and b content. Each histogram represents a mean ± SE of four independent experiments (*n* = 4). Different letters indicate significant differences between treatments (*p* < 0.05) according to Duncan’s multiple range test.

To analyze the role of cucumber TGase in salt tolerance, we overexpressed the *TGase* gene in tobacco plants. As shown in Figure 3A, as compared with WT plants, the biomass was higher in the *CsTGase*-overexpressing (*CsTGase*OE) plants after salt stress. On the other hand, the *CsTGase*OE plants had a higher biomass relative to WT plants under normal conditions. As shown in Figure 3B, the proline content in all the plants increased after salt treatment, but this increase was much greater in *CsTGase*OE plants than in the WT plants. In WT plants, the chlorophyll a and chlorophyll b contents decreased by 62.0% and 51.3%, respectively, after salt treatment, whereas they maintained higher levels in *CsTGase*OE plants compared with those of the WT (Figure 3C,D).

**Figure 4:**
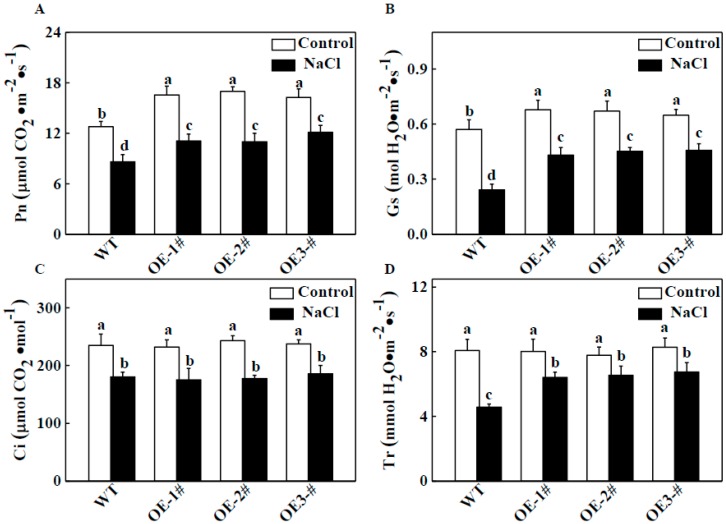
Photosynthetic parameters of WT and *CsTGase*OE plants in response to salt stress. (**A**) Net photosynthetic rate (Pn). (**B**) Stomatal conductance (Gs). (**C**) Intercellular CO_2_ concentration (Ci). (**D**) Transpiration rate (Tr). Each histogram represents a mean ± SE of four independent experiments (*n* = 6). Different letters indicate significant differences between treatments (*p* < 0.05) according to Duncan’s multiple range test.

We also evaluated the effects of salt stress on photosynthetic gas exchange parameters. As shown in Figure 4A, under normal conditions, the net photosynthesis rate (Pn) of *CsTGase*OE plants was significantly higher than that of the WT plants. Salt stress resulted in a significant decrease in Pn, but this value was still maintained at a higher level in *CsTGase*OE plants than in the WT (Figure 4A). Similar results were observed for stomatal conductance (Gs) (Figure 4B) and transpiration rate (Tr) (Figure 4D). However, there were no significant differences in intercellular CO_2_ concentration (Ci) in WT and *CsTGase*OE plants after salt stress (Figure 4C). These results suggest that TGase plays a critical role in the tobacco response to salt stress, especially in maintaining photosynthetic properties.

### 2.4. Effects of TGase on Endogenous PA Content in Thylakoid Membranes

**Figure 5:**
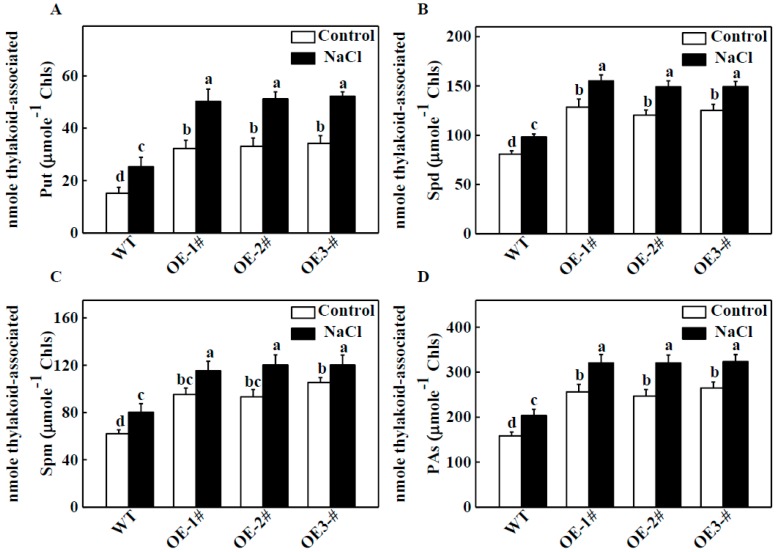
Effects of salt stress on the contents of endogenous putrescine (Put), spermidine (Spd), spermine (Spm) and total polyamines (PAs) in chloroplast of WT and *CsTGase*OE plants. (**A**) Thylakoid-associated Put content. (**B**) Thylakoid-associated Spd content. (**C**) Thylakoid-associated Spm content. (**D**) Total thylakoid-associated PAs content. Each histogram represents a mean ± SE of three independent experiments (*n* = 4). Different letters indicate significant differences between treatments (*p* < 0.05) according to Duncan’s multiple range test.

To determine whether PAs were involved in *TGase*-induced salt tolerance by protecting photosynthetic properties, we first measured the endogenous concentration of PAs in thylakoid membranes using a sensitive HPLC method. Under normal conditions, in *CsTGase*OE plants, the thylakoid associated PAs (Put, Spd and Spm) showed significantly higher levels compared with those of WT plants. Salt-induced bound Put, Spd, and Spm increased in comparison to the WT (Figure 5A–C). Meanwhile, the PA concentration increased by 161.8%, 155.9%, and 167.4% in the three *CsTGase*OE lines, respectively, compared with the WT under normal conditions (Figure 5D). PA accumulation rose by 28.7% in WT plants after salt treatment, but was still lower than in *CsTGase*OE plants.

### 2.5. Effects of TGase on the Ultrastructure of Thylakoids

**Figure 6:**
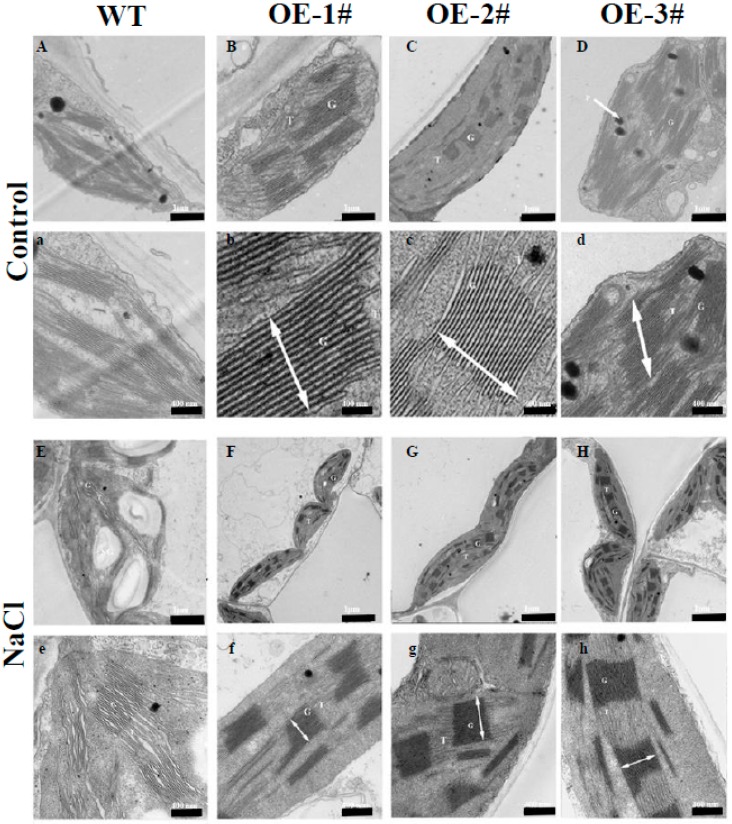
Electron microscopy in chloroplast of WT and *CsTGase*OE plants after salt stress. (**B**–**D**, **b**–**d**, **F**–**H** and **f**–**h**) shows an increased grana appression and a reduced stroma thylakoid network with respect to the WT (**A**,**a**,**E**,**e**) under normal conditions and salt stress. G, grana; T, thylakoid; SG, starch grana, P, plastoglobule. Grana height is indicated by white arrows. Scale bars for chloroplast and thylakoid are indicated. Three biological replicates were performed, and similar results were obtained.

PAs are a major positive factor in chloroplast ultrastructure [14]. To determine whether TGase regulates the ultrastructure of chloroplasts, we assayed the architecture of the thylakoid network using transmission electron microscopy (TEM). Under normal conditions, TEM revealed that the chloroplasts of WT plants had well-structured thylakoid membranes composed of grana connected by stroma lamellae (Figure 6A). Interestingly, overexpression of *TGase* resulted in chloroplasts having more grana and a larger size than those of the WT plants (Figure 6B–D). And in *CsTGase*OE plants, chloroplasts grana stacks reached up to 600 nm, whereas in the WT plants, chloroplast grana stacks were a maximum of 200 nm (Figure S1). These results suggest that TGase plays an important role in chloroplast development. Furthermore, under salt stress, chloroplasts were severely deformed into irregular shapes, and starch granules accumulated; moreover, a separation between cell membranes and chloroplasts was observed in WT plants (Figure 6E). However, the disintegration of grana thylakoids was significantly lower in *CsTGase*OE plants compared to the salt-treated WT plants (Figure 6E–H).

### 2.6. Effect of TGase on Chl a Fluorescence Transients (OJIP)

**Figure 7:**
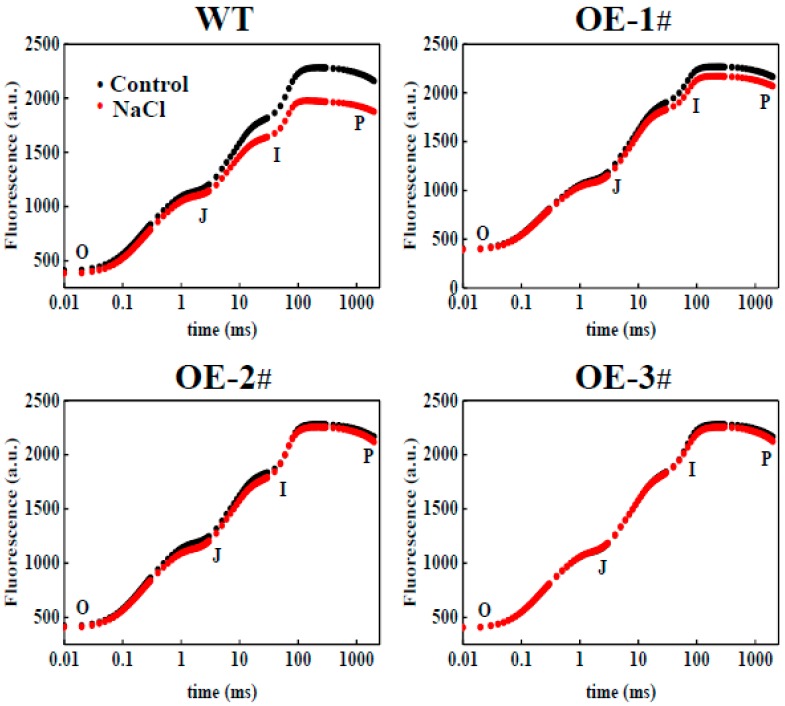
Change in Chl *a* fluorescence transient curves (OJIP) (log time scale) in leaves of WT and *CsTGase*OE plants under salt stress.

A kinetic comparison was made of the raw OJIP transients measured in *CsTGase*OE and WT plants after salt stress. No significant difference was observed under the absence of NaCl in any of the plants. However, there were significant differences in *CsTGase*OE and WT plants under salt stress (Figure 7). In WT plants, salt stress resulted in a significant decrease in the intensities of fluorescence at J, I, and P levels with no major change in the minimal fluorescence (F_0_) (Figure 7). Compared to the WT plants, the OJIP fluorescence transient and F_m_ values were near the normal levels in *CsTGase*OE plants after salt stress (Figure 7).

### 2.7. Effect of TGase on Nonphotochemical Quenching (NPQ) Induction

**Figure 8:**
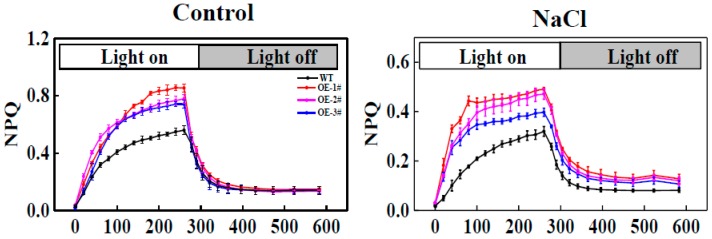
Nonphotochemical quenching (NPQ) induction and relaxation kinetics of WT and *CsTGase*OE plants under salt stress.

NPQ induction during light and dark transition periods was monitored. Transformed tobacco illuminated with 1 min light (500 μmol m^−2^ s^−1^) and treat with 4 min dark showed NPQ values proximal to ~0.8, whereas there was little activation of photoprotection in the WT, with an NPQ of ~0.4 (Figure 8A). At a light intensity level of 500 μmol m^−2^ s^−1^ the NPQ value of the WT was significantly decreased by salt stress compared to that of the *CsTGase*OE plants (Figure 8B).

### 2.8. Effect of TGase on Quantum Yield of Energy Conversion in PSII and PSI

**Figure 9:**
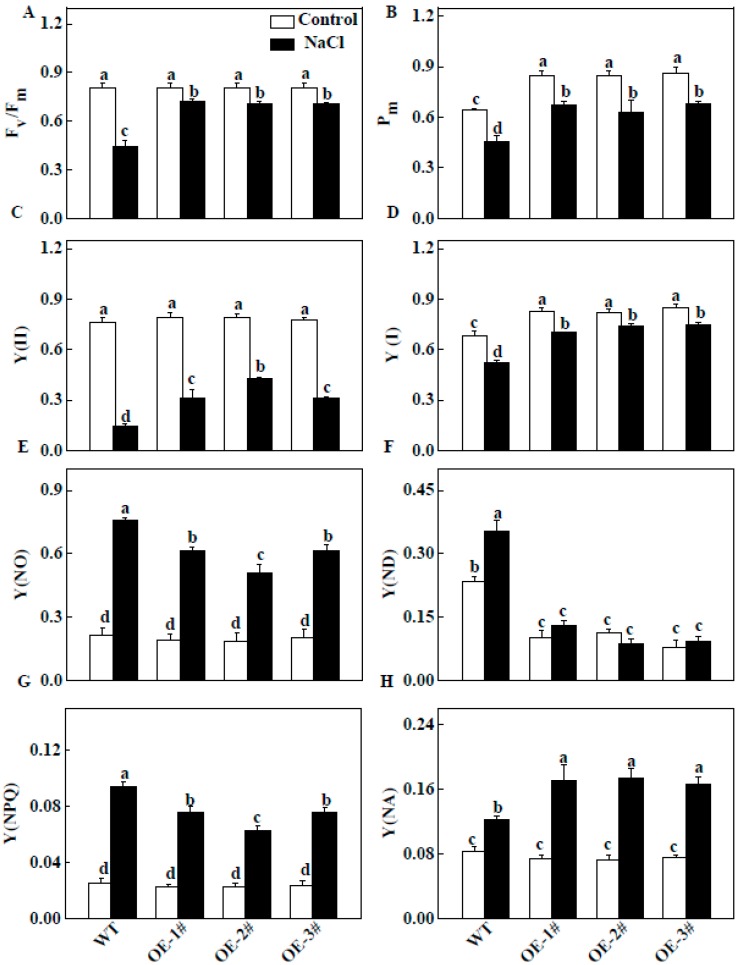
Changes in PSII and PSI in leaves of WT and *CsTGase*OE plants under salt stress. (**A**) The maximal quantum efficiency of PSII (F_v_/F_m_). (**B**) The maximum fluorescence of PSI (P_m_). (**C**) The effective quantum efficiency of PSII (Y(II)). (**D**) The effective quantum efficiency of PSI (Y(I)). (**E**) The nonregulated energy dissipation (Y(NO)). (**F**) The oxidation status of PSI donor side (Y(ND)). (**G**) The regulated energy dissipation (Y(NPQ)). (**H**) The reduction status of PSI accept or side (Y(NA)). Each histogram represents a mean ± SE of four independent experiments (*n* = 4). Different letters indicate significant differences between treatments (*p* < 0.05) according to Duncan’s multiple range test.

The F_v_/F_m_ values were not significantly different in all plants under normal conditions (Figure 9A). However, the F_v_/F_m_ values of the WT plants were significantly lower than those of *CsTGase*OE plants under salt stress (Figure 9A), indicating that photoinhibition was more severe in WT plants and the TGase has a positive role in photoprotection. The Y(II) in *CsTGase*OE plants was 19.8–24.0% higher than in WT plants, mainly due to an extremely higher photochemical quenching coefficient (qP) under normal conditions. After salt stress, the Y(NO) and Y(NPQ) values of *CsTGase*OE plants were lower compared with those of WT plants (Figure 9C–E). In *CsTGase*OE plants, the quantum yield of regulated energy dissipation (Y(NPQ)), an important process that consumes excess absorbed light energy and protects the photosynthetic apparatus, was higher than that in WT plants after salt stress (Figure 9G).

Similar to F_v_/F_m_ and Y(II), P_m_ and Y(I) increased under normal conditions and were notably higher after salt stress in *CsTGase*OE plants compared with WT plants. Y(ND) showed a significant decrease in *CsTGase*OE plants compared to that of the WT plants after salt stress (Figure 9F). In addition, Y(NA) showed a significant increase in *CsTGase*OE leaves compared with those of the WT plants after salt stress (Figure 9H).

### 2.9. Effects of TGase on the Regulation of Photosynthesis Related Gene Expression

**Figure 10:**
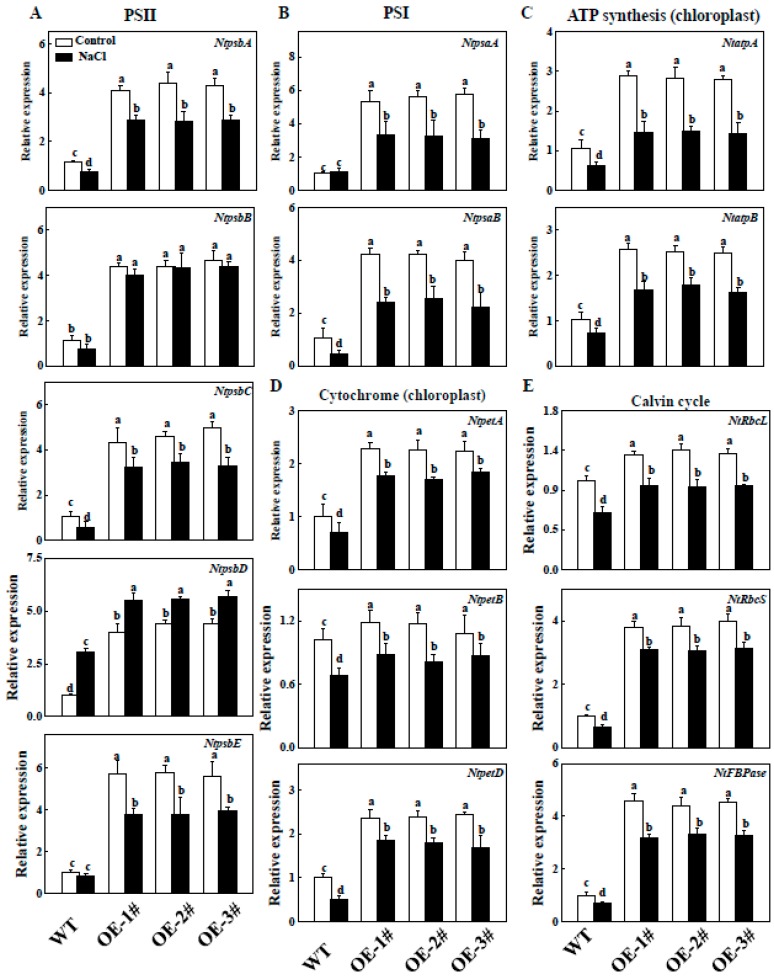
Expression of PSII (**A**); PSI (**B**); Cytochrome (**C**); ATP synthesis (**D**); Calvin cycle-related genes (**E**) in WT and *CsTGase*OE plants under salt stress. Each histogram represents a mean ± SE of four independent experiments (*n* = 4). Different letters indicate significant differences between treatments (*p* < 0.05) according to Duncan’s multiple range test.

To analyze whether TGase is involved in affecting photosynthesis-related genes, we first examined the expression of photosynthesis-related genes in the WT and *TGase*OE plants, such as PSII-related genes (*NtpsbA*/*B*/*C*/*D*/*E*), PSI-related genes (*NtpsaA*/*B*), ATP synthesis-related genes (*NtatpA*/*B*), Calvin cycle-related genes (*NtrbcL*/*NtrbcS*/*NtFBPase*) and cytochrome-related genes (*NtpetA*/*B*/*D*). As shown in Figure 10, the transcript levels of photosynthesis-related genes were higher in *CsTGase*OE plants than the WT plants under normal conditions. We further analyzed the expression patterns of those genes in WT and *CsTGase*OE plants after 7 days of salt stress. Except for *NtpsbD*, the transcript levels of these genes in WT and *CsTGase*OE plants were suppressed under salt stress, but its levels in *CsTGase*OE plants were still higher than those in the WT (Figure 10).

**Figure 11:**
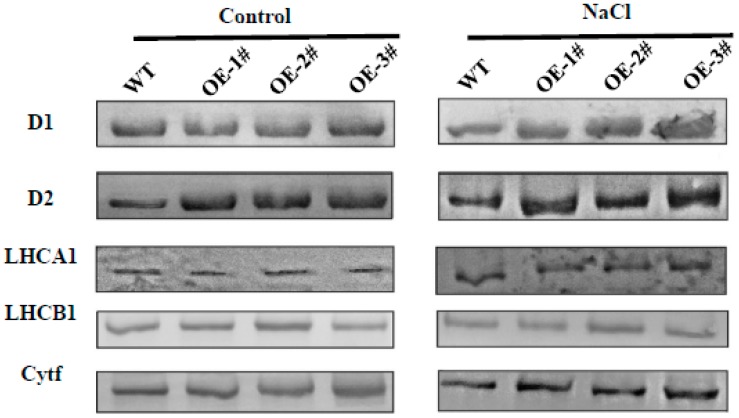
Analysis of thylakoid membrane protein changes in WT and *CsTGase*OE plants under salt stress. Thylakoid membrane proteins were separated by 12% SDS-urea-PAGE, transferred to PVDF membranes and probed with antisera against known thylakoid membrane proteins obtained from Agrisera company.

To confirm these results, we monitored the differential changes of some photosynthesis proteins using western blot (WB). As shown in Figure 11, in agreement with the results of gene expression, the levels of D2 proteins in *CsTGase*OE plants were significantly higher than that of WT plants under normal conditions. After 7 days of salt stress treatment, a significant reduction of D1, D2, and Cytf proteins was detected in the leaves of WT plants, but higher levels of these proteins were still present in the leaves of *CsTGase*OE plants (Figure 11 and Figure S2). In addition, LHCA1, and LHCB1 had showed no significant differences in WT and *CsTGase*OE plants before and after salt stress (Figure 11 and Figure S2).

## 3. Discussion

*CsTGase* expression was dependent on light induction (Figure 1A), and *CsTGase* transcript levels increased with leaf development and aging, especially in mature and senescent leaves (Figure 1B,C), supporting the notion that TGase mainly functions in the senescence process [27]. Furthermore, cucumber TGase (CsTGase) was located not only in the chloroplast grana but also in the cell wall (Figure 2). These results indicate that TGase plays a vital role in early plant development.

Salt stress and other abiotic stresses can decrease photosynthetic capacity. Under salt conditions, plants overexpressing *CsTGase* showed enhanced salt tolerance displaying vigorous growth and higher Pn and Gs (Figure 3 and Figure 4), suggesting that this gene might be particularly involved in the salt stress response. Additionally, some photosynthesis-related genes such as *NtpsbA*, *NtpsbB*, *NtpsbC*, *NtpsbD*, *NtpsbE*, *NtpsaA*, *NtpsaB*, *NtpetA*, *NtpetB*, *NtatpA,* and *NtatpB*, had higher transcript levels in *CsTGase* overexpressing plants (Figure 10), suggesting that the improved tolerance of transgenic plants over-expressing *CsTGase* might result from regulation of photosynthetic systems. Further, identification of the regulation of photosynthetic systems and unraveling this regulatory network may shed light on the mechanism underlying *CsTGase*-dependent tolerance to salt stress.

Many reports have shown that PAs play critical roles in regulating plant responses to abiotic stresses such as salinity, high temperature, and cold stresses, which are related to changes in endogenous PA levels and gene modifications [25,28,29]. *CsTGase* overexpressing lines had higher PA contents than the WT under normal conditions (Figure 5). Moreover, the overexpression of *CsTGase* increased endogenous PA levels in chloroplasts compared with those of the WT under salt stress (Figure 5), indicating that TGase may play positive roles in PA-dependent pathways to enhance plant resistance to salt stress.

The salt stress induced serious changes in photochemical efficiency and was often associated with the suppression of PSII and PSI activity [30]. In the present study, salt stress resulted in a significant decline in F_v_/F_m_ and based on the analysis of the OJIP curves, levels at J, I, and P transients were gradually decreased in WT plants under salt stress (Figure 7). This inhibition resulted in strong fluorescence and pronounced suppression of total fluorescence emission, indicating that salt stress reduced PSII and PSI electron transport. However, overexpression of *CsTGase* interrupted the decrease in the OJIP curves and F_m_ values (Figure 7). These results are consistent with the fact that TGase plays a critical role in photoprotection [31]. Electron microscopy revealed that *CsTGase* overexpression resulted in an increase in grana stacking (Figure 6). Moreover, salt stress induced more severe damage to the ultrastructure of chloroplast and thylakoids in WT plants compared with *CsTGase*OE plants. Taken together, these results indicated that TGase plays a critical role in the protection of chloroplast under salt stress.

It was demonstrated that chlorophyll-a/b proteins (LHCII, CP29, CP26, and CP24) as substrates of TGase, as well as TGase itself, can catalyze the modification of light harvesting complex II by PAs in a light-dependent pathway [32,33]. However, the regulation of PSI through TGase has not been well studied. In this study, the decrease of Y(I) in the treated WT leaves resulted from the increase in the donor side limitation of PSI, as reflected by Y(ND), whereas the Y(ND) was not increased in the *CsTGase*OE plants (Figure 9). This finding indicates that salt stress releases an excessive amount of light energy to PSI in WT plants but not in *CsTGase*OE plants. The proportion of reduced electron carriers cannot be oxidized on the acceptor side of PSI by Y(NA) in WT plants, which is often used as an indicator of PSI photoinhibition. Our results showed that TGase could alleviate the salt stress caused the acceptor side limitation of PSI, as reflected by the higher value of Y(NA) in the *CsTGase*OE plants under salt stress than in the WT plants. An increase in Y(NA) indicates an increasing acceptor side limitation [34]. This phenomenon indicates that TGase may play a positive role in PSI under salt stress.

Apart from the effect on photosystems, we also found that the Calvin cycle is regulated by *CsTGase*. Indeed, the upregulation of some Calvin cycle genes, such as *NtRbcL* and *NtRbcS,* were observed in *CsTGase*OE plants under normal conditions. Furthermore, the expression of the *NtFBPase* gene was slightly enhanced in the leaves of *CsTGase*OE plants (Figure 10). However, the expression of Calvin cycle-related genes was inhibited in WT and *CsTGase*OE plants after salt stress, but these genes still maintained a high level in *CsTGase*OE plants (Figure 10). Rubisco and FBPase are sensitive to oxidative stress, as salt stress has been shown to induce ROS accumulation [35]. Hence, we hypothesize that TGase may be involved in the downregulation of ROS accumulation by enhancing endogenous PA content, especially in chloroplasts, thereby protecting the photosynthetic organs and the Calvin cycle.

A number of previous studies have established the role of TGase in maintaining the activity of chloroplast-related proteins, which is associated with enhancement of PA conjugation to light harvesting complex II (LHCII) proteins [21]. Proteomic analysis revealed that some proteins of photosystems were substrates of TGase, such as, LHCII, CP29, CP26, and CP24 [36]. In this study, the D1 and D2 proteins were strongly accumulated in *CsTGase*OE plants, while their levels were noticeably decreased in WT plants under salt stress. In addition, the LHCA1, LHCB1 and Cytf protein levels were not significantly different in WT and *CsTGase*OE plants (Figure 11 and Figure S2). Taken together, these results suggest a critical role for TGase in maintaining protein stability under salt stress.

In summary, this study provides compelling evidence to support our assumption that TGase participates in the enhancement of salt tolerance by modulating photosynthetic proteins; the manipulation of endogenous TGase activity could increase PA levels to alleviate salt-induced photoinhibition in tobacco plants. Meanwhile, TGase increased the levels of chloroplast proteins and protected these proteins under salt stress. Therefore, TGase mediates the processing of PA biosynthesis and the protection of chloroplast proteins to enhance salt tolerance, thereby increasing the survival of plants under salt stress.

## 4. Materials and Methods

### 4.1. Cucumber Plant Materials and Treatments for Expression Analysis

The cucumber *Cucumis sativus* L. cv. 9930 genotype was used in the experiment. Seeds were germinated and grown in 250 cm^3^ plastic pots filled with peat. The plants were watered daily with Hoagland’s nutrition solution in the chamber. The growing conditions were as follows: 14/10 light/dark cycle, 28/22 °C day/night temperatures and 600 μmol m^−2^ s^−1^ photosynthetic photon flux density (PPFD).

To analyze the possible influence of light on gene expression, 30-day-old cucumber plants growing under the normal 16/8 h photoperiod, were incubated to a 24 h illumination period, with or without being previously subjected to 24 h darkness. Leaf samples were taken at 0, 4, 8, 12, and 24 h and during the continuous illumination period.

To analyze the tissue-specific expression of *CsTGase*, roots, stems, leaves, flowers and fruits were collected. Young, mature, and senescent leaves were collected. We also collected the leaves at 1 week, 2 weeks, 4 weeks, and 8 weeks during plant growth.

### 4.2. TEM Observations: Immunogold Transmission Electron Microscopy

Cucumber leaf sections were fixed, dehydrated, and embedded in Lowicryl K4M resin (Pelco International, Redding, CA, USA), following the previously described procedures [37]. A monoclonal antibody, anti-plant TGase (produced in rabbit; Univ-bio, Shanghai, China) at 1:1000 dilutions was used. A gold AffiniPure anti rabbit IgG was used as a secondary antibody. For electron microcopy, a Jeol-JEM-1010 transmission electron microscope operating at 80 kV.

### 4.3. Generation and Selection of Transgenic Plants

To obtain the cucumber *TGase* overexpression (*TGase*OE) construct, the 1836 bp full-length coding DNA sequence (CDS) was amplified with the primer *TGase*OE-F (5′-CGAGCTCATGGATGATCGTGAGGCGTTTAAGA-3′) and *TGase*OE-R (5′-CGGGGTACCACGTTGCATGCAATTCCCGTAG-3′) using cucumber cDNA as the template. The PCR product was digested with *Sac*I and *Kpn*I and inserted behind the CaMV 35S promoter in the binary vector pCAMBIA1301-GUS. The *TGase*OE-GUS plasmid was transformed into *Agrobacterium tumefaciens* strain EHA105. NC89 tobacco plants were used for transformation, as described by Horsch [38].

### 4.4. Salt Tolerance Analysis of the Transgenic Plants

Three-week-old plants grown in vermiculite were treated with 200 mM NaCl for 7 days, and leaves were used to measure the biomass, proline content, and chlorophyll content. For biomass measurements, plants were dried for 48 h at 75 °C and then weighed. The proline content was measured according to Bates et al. [39]. The chlorophyll content was measured by UV spectrophotometry as described by Yang et al. [40].

Three-week-old plants grown in vermiculite were treated with 200 mM NaCl for 14 days, and the leaves were used to measure the photosynthetic parameters. The net photosynthetic rate (Pn), intercellular CO_2_ concentration (Ci), and stomatal conductance (Gs) were measured according to Zhang et al. [41].

To avoid light impact on the TGase activity and PA content, potted plants in the experimental field were exposed to a 14/10 light/dark cycle. Then, we collected the samples and measured photosynthetic parameters at 12 h of illumination.

### 4.5. Thylakoid Isolation

Thylakoids were isolated as previously described with minor modifications [13]. Intact chloroplasts from the fully expanded leaves were homogenized in 50 mM KCl, 1 mM MgCl_2_, 1 mM MnCl_2_, 1 mM EDTA, 0.5 mM KH_2_PO_4_, 25 mM HEPES, pH 7.6, 330 mM sorbitol, 10 μM sodium ascorbate, and 0.2% (*w*/*w*) bovine serum albumin. The homogenates were filtered through a 300-μm and then 100-μm nylon mesh and debris was removed by centrifugation at 300× *g* for 1 min, followed by centrifugation at 4000× *g* for 10 min to collect the thylakoids. The pellet was separated from starch, resuspended and washed in 7 mM MgCl_2_, 10 mM KCl, and 25 mM HEPES, pH 7.6 to break intact chloroplasts and removed free polyamines. Finally, for the polyamine analysis, thylakoids were resuspended in the medium containing 7 mM MgCl_2_, 50 mM KCl, 25 mM HEPES, pH 7.6 and 330 mM sorbitol.

### 4.6. Analysis of Endogenous Polyamines in Thylakoid Membranes

The contents of endogenous PAs in thylakoid membranes were analyzed according to a method described by Zhang et al. [41] with some modifications. Briefly, for polyamine analysis, isolated thylakoids were incubated in 1.6 mL of 5% (*w*/*v*) cold perchloric acid (PCA) for 1 h on ice. After centrifugation for 20 min at 12,000× *g*, the pellet was used to determine bound PAs. PAs were analyzed using a high-performance liquid chromatography with a 1200 series system (Agilent Technologies, Santa Clara, CA, USA), a C18 reversed-phase column (4.6 mm by 250 mm, 5 μm Kromasil) and a two solvent system including a methanol gradient (36%–64%, *v*/*v*) at a flow rate of 0.8 mL min^−1^.

### 4.7. Observation of the Ultrastructure of the Chloroplast

Tobacco leaves were collected and cut into pieces of approximately 1 mm^2^ and fixed by vacuum infiltration with 3% glutaraldehyde and 1% formaldehyde in a 0.1 M phosphate buffer (pH 7.4) for 2 h (primary fixation). After washing, the sample were fixed for 2 h in osmium tetroxide at room temperature; then, the samples were dehydrated in acetone and embedding in Durcupan ACM, Ultrathin sections of the leaf pieces (70 nm) were cut, stained with uranium acetate and lead citrate in series and examined using a H7650 transmission electron microscope (Hitachi, Tokyo, Japan) at an accelerating voltage of 80 kV. A minimum of 50 chloroplasts of each type of plant were examined.

### 4.8. Chl a fluorescence Measurement and OJIP Transient Analyses

The chlorophyll *a* (Chl *a*) fluorescence induction kinetics were measured at 12 h of illumination using dual portable fluorescence (Dual-PAM-100, Walz, Germany). Measurements were analyzed using the automated induction program provided by the Dual-PAM software. PSII and PSI activities were quantified by chlorophyll fluorescence and P700^+^ absorbance changes.

The OJIP curves showed a polyphasic rise. The initial fluorescence (F_0_) (approx. 50 μs) was set as O, followed by the O to J phase (ends at approx. 2 ms), then the J to I phase (ends at approx. 30 ms) and I to P phase (at the peak of the OJIP curve). The JIP measurement is named after the basic steps in fluorescence transience when plotted on a logarithmic time scale [42]. Leaves were dark adapted for 30 min prior to the measurement or the NPQ measurement leaves were continuously illuminated for 270 s with 500 μmol photons m^−2^ s^−1^ using the Handy-PEA (multihit- mode). Every 30 s was given a 3000 μmol photons m^−2^ s^−1^ (duration 0.8 s) saturating pulse for maximal fluorescence, F_m_′. To calculate the NPQ at the end of the actinic light phase we followed the equation, NPQ = F_m_/ F_m_′ − 1 [43]. The tests were shown in the middle portion of infiltration on the ventral surface of the leaves. Measurements were taken in 6 replications. 

### 4.9. Quantitative Real-Time PCR

Total RNA was extracted from three biological replicates of leaves using an RNA extraction kit (Tiangen, Beijing, China) according to the manufacturer’s instructions. The first strand cDNA was synthesized from 1 μg of DNase-treated RNA using reverse transcriptase (Takara, Dalian, China) following the manufacturer’s protocol. Quantitative real-time PCR (qPCR) was using gene specific primers (Table S1) in 20 μL reaction system using SYBR Premix Ex Taq II (Takara, Dalian, China). Tobacco *β-actin* was used as a reference gene for tobacco; cucumber *actin* was used as a reference gene for cucumber. Relative gene expression was calculated according to Livak and Schmittgen [44].

### 4.10. Protein Extraction and Western Blotting

For the thylakoid membranes, the intact chloroplasts were reputed in low osmotic buff (50 mM HEPES–KOH (pH 7.6) and 2 mM MgCl_2_) on the ice, then the thylakoid membranes were collected and the protein content was determined by a BCA Protein Assay Kit (Solarbio, Beijing, China). For immunoblot analysis, thylakoid proteins were solubilized and separated on 12% SDS-urea-PAGE gels. After electrophoresis, the proteins were transferred to polyvinylidene difluoride (PVDF) membranes (Millipore, Billerica, MA, USA) and probed using commercial antibodies specific for the PSII subunits (D1 and D2) (AS05084 and AS06146), light-harvesting antenna proteins (LHCA1 and LHCB1) (AS01005 and AS01004) and Cytb6f subunit (Cytf) (AS14169), at 1:5000 dilutions were used. These antibodies were from Agrisera (Vännäs, Sweden). At least three independent replicates were used for each determination. Accumulation of proteins were quantified using Quantity One software (Bio-Rad, Hercules, California, CA, USA).

### 4.11. Statistical Analysis

At least 4 independent replicates were used for each determination. Statistical analysis of the bioassays was performed using the SPSS 20 statistical package (SPSS Inc., Chicago, IL, USA). Experimental data were analyzed with a Duncan’s multiple range test at *p* < 0.05.

## Supplementary Materials

Supplementary materials can be found at http://www.mdpi.com/1422-0067/20/4/894/s1. Figure S1: The granum height of TGase in each transgenic line. Figure S2: The relative intensity of thylakoid membrane protein changes in WT and *CsTGase*OE plants under salt stress. Table S1: Sequences of primers used for qPCR assays.
