# Supplementary material for: Overexpression of Transglutaminase from Cucumber in Tobacco Increases Salt Tolerance through Regulation of Photosynthesis
Source: Int J Mol Sci. 2019 Feb 19;20(4):894. doi: 10.3390/ijms20040894 (PMC6413182; doi:10.3390/ijms20040894)
Supplement: Supplementary file 1 [file ijms-20-00894-s001.pdf]

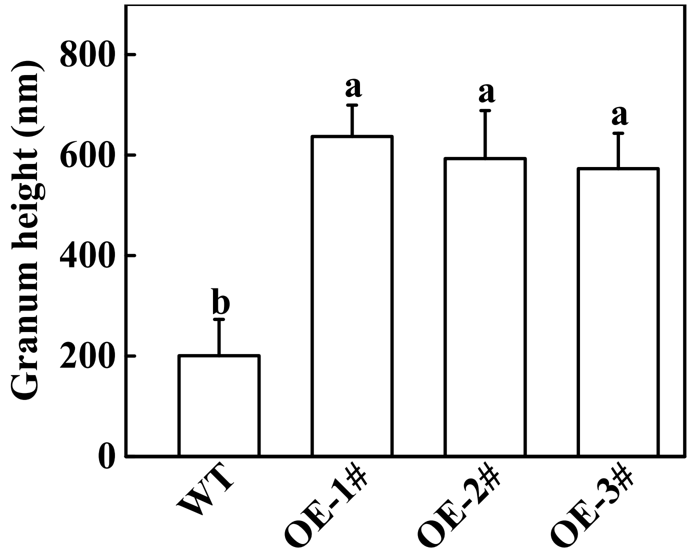

Figure S1. The granum height of *TGase* in each transgenic line. Each histogram represents a mean± SE of four independent experiments (n=4). Different letters indicate significant differences between treatments (*P*<0.05) according to Duncan's multiple range tests.

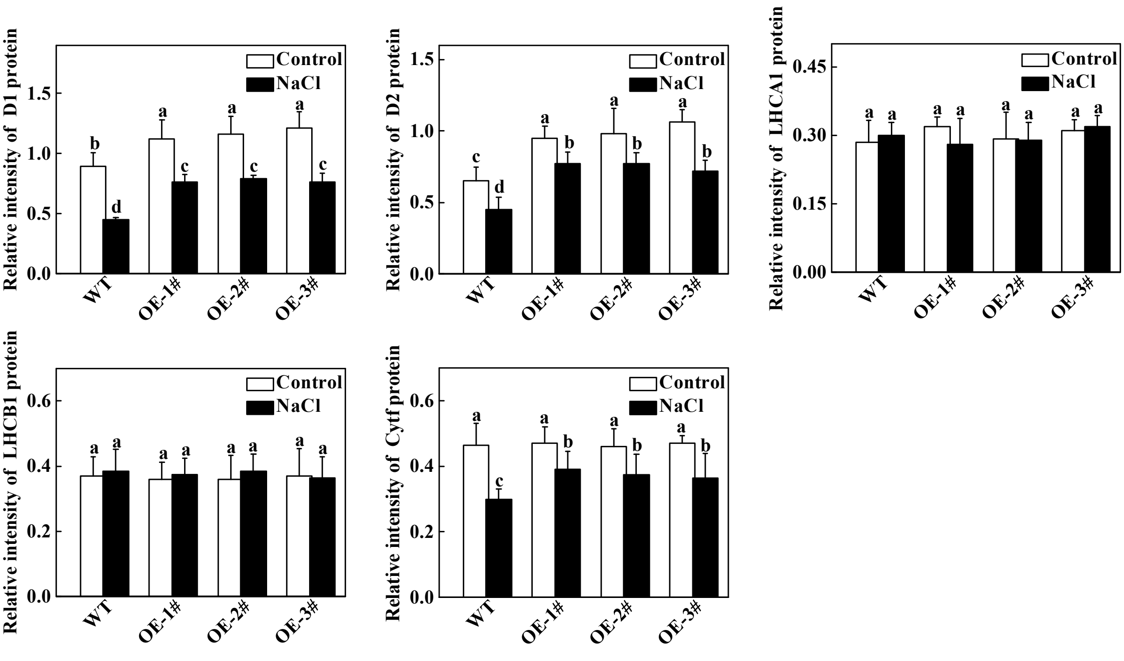

Figure S2. The relative intensity of thylakoid membrane protein changes in WT and *CsTGase*OE plants under salt stress. Each histogram represents a mean± SE of three independent experiments (n=3). Different letters indicate significant differences between treatments (*P*<0.05) according to Duncan's multiple range tests.

Supplemental Table 1. Sequences of primers used for gene expression analysis.

Gene name	Forward primer (5′ to 3′)	Reverse primer (5′ to 3′)
NtpsbA	CCATCTACAAATGGATAAGATCCTG	AGCAATAGCACCCTCTTGATAGAA
NtpsbB	TTTGCTGGTATTGACCCAGA	GGGCGAAAGAAAGATACCAA
NtpsbC	TGAGATGAGACAGGAGATCCAAT	GCTATCCGAAAGAAAAGGGAAT
NtpsbD	GCGGAAGATCCTGAATTTGA	CCACGTGGTAGAACCTCCTC
NtpsbE	AGCACCGGTTTAGCTTACGA	AGGGCCTCCTAAAACGATCT
NtpsaA	GTGCTGTAGGAGTAACCCATTACC	CAAATCCTCCTAGCCATTATCCTA
NtpsaB	CTAGGATTCGACTTGTATCATGGA	GACGGAACCTTGCTTATTTCTTTC
NtatpA	GAAGCTATTCAGGAACAAATGGAC	AATCCAAGACGCTTGATTCCTT
NtatpB	TACCAAATCACGCCCCTATT	AGCAAAACCACCCATCAGAG
NtRbcL	GTCCCCTGTTGGGATGTACTATT	TGTGAGTTCACGTTCTCATCATC
NtRbcS	TCATTGGATTCGACAACGTG	CACAACCCCTAAAGACAAGACA
NtFBPase	TCGGAAAACCAAGGTGAAAG	AGCCATTGGTACCATCCTAGA
NtpetA	ATTTGGTCAGGGAGATGCAG	TGTCGACAAATCTGCGAATC
NtpetB	CCGCTTCTTACTGCCGTATT	TCCTCCACCAAGTGATAAATGA
NtpetD	TTGCAACCCCTTTGGAAATA	CCGCAGGTACTGAAACCATT
Ntactin	CATTGGCGCTGAGAGATTCC	GCAGCTTCCATTCCGATCA
CsTGase	GGAGATACAACAACATATCAAGA	TTCAGTAAGCCATATAGTTCAAC
Csactin	CAGGAATCCACGAAACTACT	AGACCCTCCAATCCAAACAC
